# Probing for consciousness in machines

**DOI:** 10.3389/frai.2025.1610225

**Published:** 2025-08-20

**Authors:** Mathis Immertreu, Achim Schilling, Andreas Maier, Patrick Krauss

**Affiliations:** ^1^CCN Group, Pattern Recognition Lab, Erlangen, Germany; ^2^University Erlangen-Nürnberg, Erlangen, Germany; ^3^University Hospital Mannheim, Mannheim, Germany; ^4^University Heidelberg, Heidelberg, Germany; ^5^Neuroscience Lab, University Hospital Erlangen, Erlangen, Germany

**Keywords:** machine consciousness, reinforcement learning, core consciousness, self model, world model, virtual environment, probes

## Abstract

This study explores the potential for artificial agents to develop core consciousness, as proposed by Antonio Damasio's theory of consciousness. According to Damasio, the emergence of core consciousness relies on the integration of a self model, informed by representations of emotions and feelings, and a world model. We hypothesize that an artificial agent, trained via reinforcement learning (RL) in a virtual environment, can develop preliminary forms of these models as a byproduct of its primary task. The agent's main objective is to learn to play a video game and explore the environment. To evaluate the emergence of world and self models, we employ probes–feedforward classifiers that use the activations of the trained agent's neural networks to predict the spatial positions of the agent itself. Our results demonstrate that the agent can form rudimentary world and self models, suggesting a pathway toward developing machine consciousness. This research provides foundational insights into the capabilities of artificial agents in mirroring aspects of human consciousness, with implications for future advancements in artificial intelligence.

## Introduction

In light of the increasing capabilities and presence of intelligent systems in our daily lives, determining whether machines can become conscious is an increasingly urgent question. Moreover, the exact nature of consciousness remains an unsolved problem, with various contradictory theories about its origins and mechanisms.

As modern computers emerged and their capabilities grew, the idea of machines becoming conscious was already being contemplated, alongside the challenge of how such a condition might be evaluated. One of the earliest and most well-known proposals in this context is the Turing Test (Turing, 1950), in which a human judge interacts with an unknown partner—either another human or a machine. If the machine's responses are indistinguishable from a human's, it is said to have passed the test. While often cited in discussions of artificial consciousness, it is important to note that Turing originally framed this test as a criterion for intelligence, not for consciousness. Nonetheless, some modern large language models (LLMs), starting with GPT-4, have arguably passed this behavioral threshold of indistinguishability (Jones and Bergen, 2024).

However, does this behavioral success imply that such models are conscious? John Searle's famous Chinese Room thought experiment (Searle, 1980) challenges this notion. In the scenario, a person who does not understand Chinese manipulates symbols using a rule book to produce valid responses in Chinese, creating the illusion of understanding. Yet, there is no semantic comprehension—only syntactic manipulation. This illustrates a key limitation of behavioral tests: sophisticated output does not guarantee internal understanding or conscious awareness.

While Searle's critique is primarily directed at claims of understanding, not consciousness per se, it underscores the broader concern that externally observable behavior may not reveal what internal processes, if any, are taking place. This issue becomes even more salient when considering artificial consciousness, where internal access is limited and the risk of over-attribution is high. As discussed in the article “Understand AI Sentience? First Understand It in Animals” (Andrews and Birch, 2023), attributing consciousness based on performance alone is especially problematic in systems that lack biological grounding.

This distinction between external behavior and internal representation lies at the heart of many debates in consciousness research. Does behavioral mimicry imply genuine understanding or awareness? More targeted proposals, such as the so-called Garland Test, aim to evaluate artificial consciousness directly by probing for internal models, self-awareness, and subjective-like responses. Our work follows this more structural line of inquiry: rather than assessing agents purely on behavior, we investigate whether reinforcement learning agents trained in virtual environments can develop internal models that resemble those hypothesized to be necessary for consciousness.

Among the prominent theories of consciousness, three stand out for their influence and relevance to artificial systems: Integrated Information Theory (IIT) (Tononi et al., 2016), Global Workspace Theory (GWT) (Baars, 2013), and Damasio's model of consciousness (Damasio and Damasio, 2022; Damasio and Meyer, 2009; Man and Damasio, 2019). IIT conceptualizes consciousness as the capacity of a system to integrate information, quantified by a measure called Φ. While theoretically rigorous, IIT's reliance on full system causal modeling makes it challenging to implement in scalable machine learning systems. GWT, in contrast, proposes that consciousness arises when information becomes globally available to multiple cognitive processes via a central workspace, often linked to attentional control and symbolic reasoning. However, typical reinforcement learning agents lack the architectural mechanisms—such as attention-based competition or global broadcasting—needed to instantiate GWT faithfully. Damasio's theory of consciousness stands apart from many mainstream accounts by emphasizing subcortical and embodied processes, in particular by emphasizing the role of bodily states, emotions, and homeostatic regulation in generating a core self through the integration of self- and world models. Crucially, its layered structure (protoself, core consciousness, extended consciousness) aligns well with the embodied, goal-directed nature of RL agents, offering a conceptually grounded yet computationally tractable framework for exploring the emergence of conscious-like representations in artificial systems.

**Figure 1:**
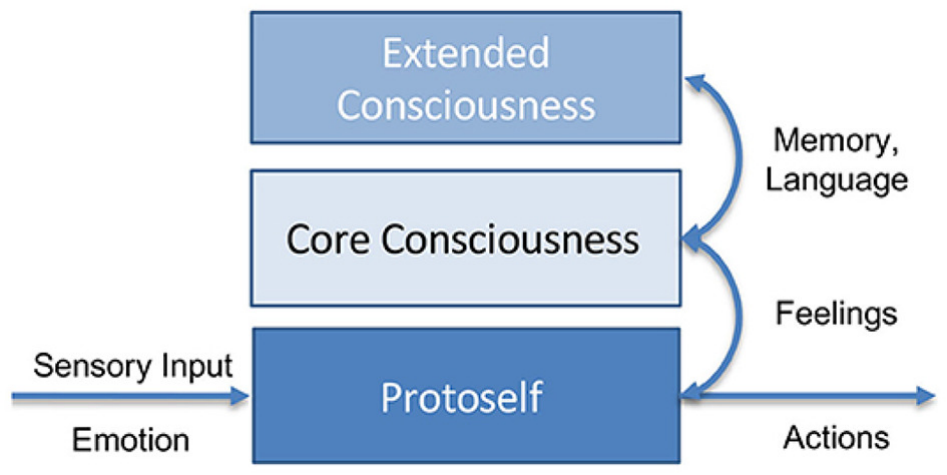
Simplified representation of Damasio's model of consciousness (taken from Krauss and Maier, 2020). The protoself operates at an unconscious level, processing emotions and sensory input. Core consciousness emerges from the protoself, creating the initial self and world models, allowing the self to relate to its environment. Projections of emotions evolve into higher-order feelings. With access to memory and the integration of complex functions such as language processing, extended consciousness develops, further enhancing the self and world models.

Moreover, only Antonio Damasio's theory of consciousness (Damasio and Meyer, 2009) provides a detailed mechanistic explanation of how consciousness arises, its attributes, and its possible embodiment in the human brain. In Krauss and Maier (2020) it is argued that this theory is uniquely well-suited for application to artificial intelligence (AI) and machine learning (ML) systems. Damasio structures consciousness into three hierarchical levels: Firstly, the protoself, the neural representation of the body state. Secondly, the core consciousness, a higher-level representation of the self, the world, and their mutual relations, leading to a transient core self, and thirdly, the extended consciousness, which includes memory, language, planning, and other high-level mental activities enabling the continuous autobiographic self. In this theory, emotions and feelings play a crucial role. Emotions are unconscious reactions to stimuli, and feelings are neural representations of these emotions. Objects can induce emotions, leading to changed feelings and a changed protoself. The combined neural representation of the perceived object and the changes in the protoself due to it forms the core consciousness and creates a core self, a sense of perception belonging to oneself. The autobiographic self, built on top of the core self, includes memories of one's past, consistent characteristics, and future plans. This requires extended consciousness and its functions like memory and planning. From simple reactive emotions to complex plans enabled by extended consciousness, these systems aim to regulate homeostasis, keeping the internal state in a safe range to ensure continued existence and increase the chance of survival. A simplified overview of Damasio's model is illustrated in Figure 1.

The concept of emotions, and thus the entire theory, can be applied to AI systems as reactive changes in their embodiment due to stimuli. For robots, this could mean changes in battery levels, actuator positions, or angles, while for smart factories, it might involve changes in cooling systems or production facilities. In computer games, which simulate the world, the agent has a simulated body. Depending on the game, changes in hit points, levels, attributes, resources, or scores can be considered emotions.

This framework allows us to assess whether an AI meets the criteria for different levels of consciousness. Krauss and Maier (2020) argued that modern algorithms might already be close to achieving “core consciousness.” Emotions that move the internal state of the embodiment toward or away from an optimal region can be seen as positive or negative emotions, aligning with the concept of regulating homeostasis. This translates directly to positive and negative rewards in reinforcement learning (RL). RL has demonstrated considerable success in training robots (Gu et al., 2017), machines (Degrave et al., 2022), and game agents (Mnih et al., 2013), making it a natural choice for training embodied AI systems based on emotions.

In applying Damasio's theory to artificial systems, one key advantage lies in the accessibility of the agent's internal processing: within the reinforcement learning architectures used in this study, all “brain” activity—such as neural activations, hidden states, and memory traces—is fully observable and can be systematically manipulated. This level of transparency enables fine-grained analysis of internal neural representations^1^ and their functional relevance to behavior. We note, however, that this accessibility is specific to the relatively compact and controlled agent architectures used here. In contrast, large-scale systems such as LLMs (e.g., GPT-4) are often treated as black boxes due to their vast parameter spaces and emergent behaviors, making it considerably more difficult to trace or interpret their internal dynamics.

We can increasingly narrow down our research questions to testable hypotheses:

1. Can a machine become conscious?
2. Can a machine possess core consciousness as defined by Damasio?
3. Can a machine develop models of the world, itself, and their mutual relations?
4. Can an agent in a computer game develop models of the game environment, itself, and their mutual relations?

In this context, a world model refers to a neural network that maps external perceptions to an internal/neural representation containing essential structures and dynamics^2^ for the agent. A self model similarly processes perceptions of its internal state. A model sufficient for core consciousness must include both and further model their relationship, such as the boundary between the self and the external world and how changes in the world affect the self and vice versa.

**Figure 2:**
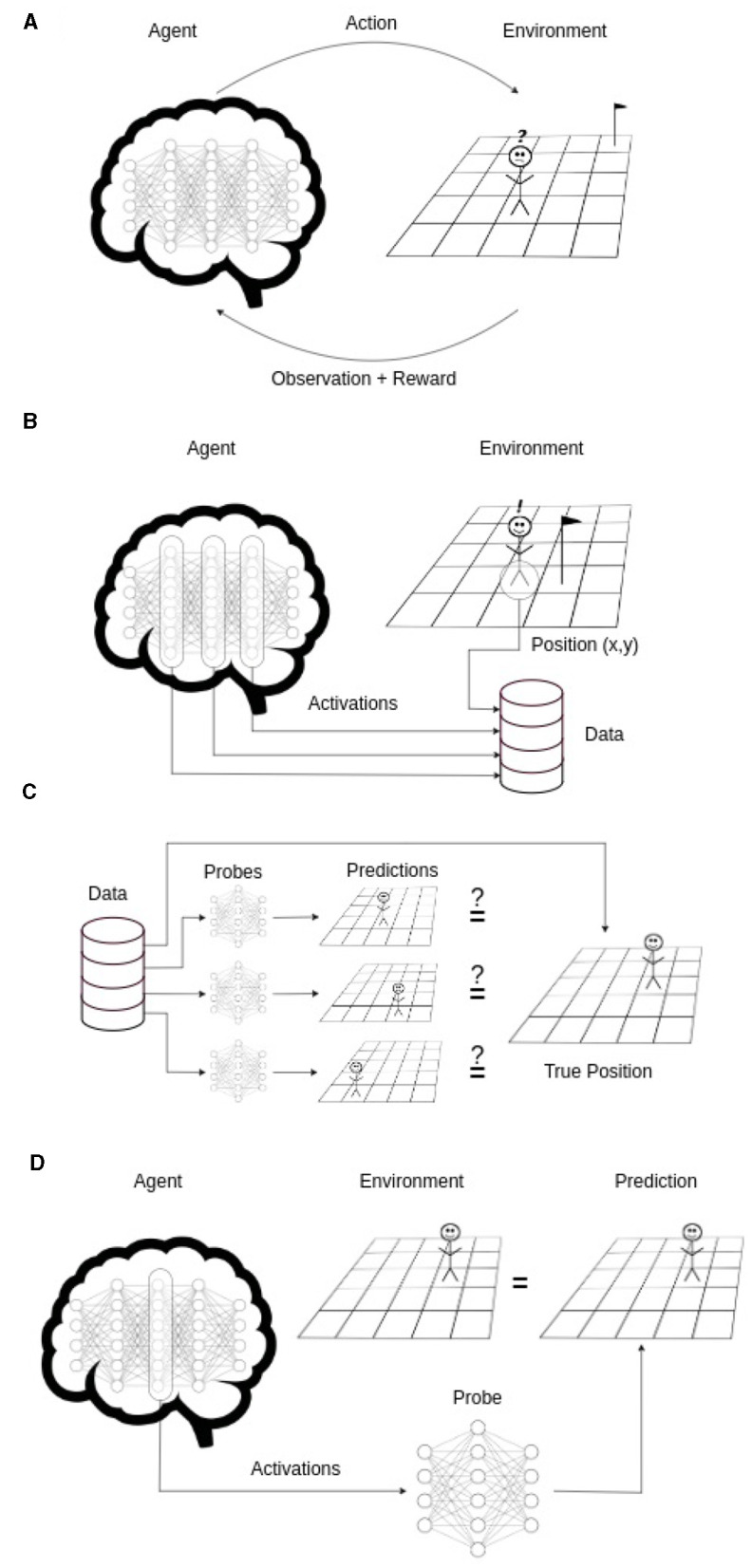
Schematized overview of the approach. **(A)** An agent is trained with RL. **(B)** A dataset of the trained agent's position and neural network's activations is sampled. **(C)** Using this dataset on each layer's activations a probe is trained to predict the true position. **(D)** If one of the probes can predict the true agent position (with an accuracy significantly higher than chance), it shows that the necessary information is contained in the activations. Thus the agent developed a world model.

In Li et al. (2022) it is demonstrated that a transformer trained on sequences of Othello moves developed a world model, effectively modeling the entire board state. Following this idea, we train probes, small classifiers, on the activations of the trained agent to determine if it understands its position in the world. The approach is summarized in Figure 2.

## Methods

###  Reinforcement learning

**Figure 3:**
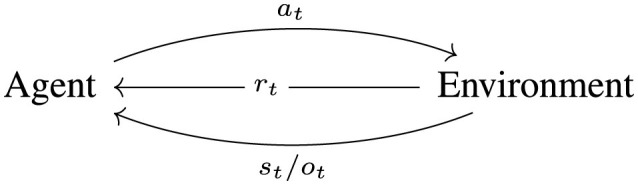
The basic agent-environment interaction cycle. The agent observes the current state/observation *s*_*t*_/*o*_*t*_, decides on an action *a*_*t*_ based on its policy and the environment reacts to this action by returning a reward *r*_*t*_ and the next state/observation *s*_*t*+1_/*o*_*t*+1_ beginning the next cycle.

Reinforcement Learning (RL) tries to solve the problem of (optimal) sequential decision making. The basic framework assumes that at every time step *t* ∈ ℕ_0_ the agent acts on the environment with an action *a*_*t*_ ∈ *A* and the environment returns a state/observation *s*_*t*_/*o*_*t*_ ∈ *S*/*O* and a reward *r*_*t*_ ∈ ℝ to the agent based on transition probabilities $P\left(S_{t+1}=s^{\prime }|S_{t}=s,A_{t}=a\right)$ and a reward function *R*:*S*×*A* → ℝ. *A* denotes the action space, *S* the state space and *O* the observation space, which depend on the chosen environment and agent. They can be either continuous or discrete, but for simplicity we focus on the discrete case and assume an episodic setting. The agent learns via this feedback loop to improve its behavior. The basic RL cycle is illustrated in Figure 3.

This notion can be further formalized as a (partially observable) Markov decision process. For this we refer to the literature on RL e.g., (Sutton and Barto 2018).

The actions of an agent are characterized by a policy which can be either deterministic


$$\begin{array}{l} \pi :S\to A\text{ via}\pi \left(s\right)=a \end{array}$$

or probabilistic


$$\begin{array}{l} \pi :S\to {\left[0,1\right]}^{\left|A\right|}\text{via }\pi \left(a|s\right)=P\left(A=a|S=s\right). \end{array}$$

We denote a policy realized as neural network with weights θ as π_θ_.

In this setting the objective is to maximize the expected return, i.e. the expected discounted cumulative reward achieved in an episode,


(1) $$\begin{array}{l} J\left(\pi_{\theta }\right):=E_{\pi_{\theta }}\left(R\left(\tau \right)\right)=\underset{\tau \in \tau }{\sum }P\left(\tau |\theta \right)R\left(\tau \right) \end{array}$$

with


(2) $$\begin{array}{l} R\left(\tau \right):=\overset{T}{\underset{i=0}{\sum }}\gamma^{i}R\left(s_{t},a_{t}\right) \end{array}$$

for trajectories


$$\begin{array}{l} \tau =\left(s_{0},a_{0},r_{0},s_{1},a_{1},r_{1}...,s_{T+1}\right)\in \tau , \end{array}$$

the space of trajectories, a discount factor γ ∈ [0, 1] and a reward function *R*:*S*×*A* → ℝ with the probability


(3) $$\begin{array}{l} \;P\left(\tau |\theta \right):= \end{array}$$

$$\begin{array}{l} \rho \left(s_{0}\right)\prod_{t=0}^{T}P\left(S_{t+1}=s_{t+1}|S_{t}=s_{t},A_{t}=a_{t}\right)\pi_{\theta }\left(a_{t}|s_{t}\right), \end{array}$$

starting state distribution ρ and episode length *T*.

The discount factor γ < 1 ensures mathematical convergence for *T* = ∞, meaning that the sum of discounted future rewards converges to a finite value even when considering an infinite horizon. This discount factor can be interpreted as modeling uncertainty about the future, as it reduces the impact of rewards that are further away in time, effectively placing more weight on immediate rewards. As a result, the agent prefers closer and more certain rewards compared to those that are further and more uncertain.

In RL, approaches to optimize the objective defined in Equation 1 can be categorized into model-based and model-free. In model-based RL, a model of the environment is given or learned e.g. modeling transition dynamics and rewards. This model can be used to directly optimize the objective with planning algorithms like MCTS (Chaslot et al., 2008) as has been successfully used with a given model in AlphaGo (Silver et al., 2016) or with a learned model in MuZero (Schrittwieser et al., 2020). The second way is to use the model as a simulator to train a model-free agent on the environment model instead of the real environment e.g. as in dreamer (Hafner et al., 2023) or world model Ha and Schmidhuber, (2018). Model-free RL can be further divided into policy optimization and Q-learning based algorithms. Policy optimization algorithms directly optimize the policy e.g., by gradient ascent as in Reinforce (Sutton et al., 1999). Q-Learning algorithms estimate the value of each state-action pair and a policy can be derived e.g., by acting (epsilon) greedy on these value estimates as in DQL (Mnih et al., 2013). Combining these two ideas leads to the class of actor-critic algorithms such as PPO (Schulman et al., 2017) or SAC (Haarnoja et al., 2018). Model-based approaches are more sample efficient and computationally intensive and model-free approaches, especially policy-based ones, are sample inefficient, but require much less computation as discussed e.g. in Gao and Wang (2023). Different exploration strategies enhance an agent's ability to explore the environment and encounter novel situations. Entropy-based methods sample actions from an action distribution and regularize the distribution's entropy to avoid premature collapse (Williams and Peng, 1991). Prediction-based exploration measures the novelty of a state and adds an exploration reward based on it, involving predicting forward dynamics (Schmidhuber, 1991), inverse dynamics (Pathak et al., 2017), or random features (Burda et al., 2018). In memory-based exploration, the agent remembers interesting states and attempts to reach them again (Ecoffet et al., 2019).

#### Proximal policy optimization

In PPO the objective *J* defined in Equation 1 is improved via gradient ascent. If the state transition dynamics and reward function were known, gradient ascent could be applied directly, but since this is usually not the case the (policy) gradient


(4) $$\begin{array}{l} \nabla_{\theta }J\left(\pi_{\theta }\right)=E_{\pi_{\theta }}\left(\overset{T}{\underset{t=0}{\sum }}\nabla_{\theta }\log\pi_{\theta }\left(a_{t}|s_{t}\right)G_{t}\right) \end{array}$$

with the reward to go


(5) $$\begin{array}{l} G_{t}=\overset{T}{\underset{i=t}{\sum }}\gamma^{i-t}R\left(s_{t},a_{t}\right) \end{array}$$

needs to be estimated from sampled trajectories. The simplest way is to use a Monte Carlo estimate


(6) $$\begin{array}{l} {Ĝ}_{t}=\overset{T}{\underset{i=t}{\sum }}\gamma^{i-t}r_{i}. \end{array}$$

This is an unbiased estimate, but has very high variance. To reduce the variance, a baseline can be subtracted and an estimator of the expected return trained, although the estimation induces some bias. The choice in PPO is to estimate the value function (represented as neural network)


(7) $$\begin{array}{l} V_{\pi_{\theta }}\left(s\right):=E_{\pi_{\theta }}\left(\overset{T}{\underset{k=t}{\sum }}\gamma^{k-t}R\left(s_{t},a_{t}\right)|S_{t}=s\right). \end{array}$$

The value function is usually trained with an n-step Bellman equation


(8) $$\begin{array}{l} {\hat{V}}_{\pi_{\theta }}\left(s_{t}\right):=\left(\overset{n-1}{\underset{i=0}{\sum }}\gamma^{i}r_{i}\right)+{\hat{V}}_{\pi_{\theta }}\left(s_{t+n}\right). \end{array}$$

The Bellman equation (Bellman, 1954) defines a fix point operator and it can be shown, that it is guaranteed to converge for linear approximators (Melo and Ribeiro, 2007), but not necessarily for non-linear ones as demonstrated in Tsitsiklis and Van Roy (1997). The value function is used to estimate the advantage function


(9) $$\begin{array}{l} \;A_{\pi_{\theta }}\left(s_{t},a_{t}\right):= \end{array}$$

$$\begin{array}{l} E_{P\left(S_{t+1}=s_{t+1}|S_{t}=s_{t},A_{t}=a_{t}\right)}\left(R\left(s_{t},a_{t}\right)+\gamma V_{\pi_{\theta }}\left(s_{t+1}\right)\right)-V_{\pi_{\theta }}\left(s_{t}\right), \end{array}$$

which indicates how much better or worse an action is compared to the mean and can be generalized to take into account multiple future steps via the generalized advantage estimator (Schulman et al., 2015)


(10) $$\begin{array}{l} {Â}_{\pi_{\theta }}\left(s_{t},a_{t}\right):=\delta_{t}+\left(\lambda \gamma \right)\delta_{t+1}+\cdots +{\left(\lambda \gamma \right)}^{T-t}\delta_{T} \end{array}$$

with parameter λ ∈ [0, 1] and


(11) $$\begin{array}{l} \delta_{t}=r_{t}+\gamma {\hat{V}}_{\pi_{\theta }}\left(s_{t+1}\right)-{\hat{V}}_{\pi_{\theta }}\left(s_{t}\right) \end{array}$$

setting the value estimate of terminal states ${\hat{V}}_{\pi_{\theta }}\left(s_{T+1}\right)=0$. This leads to the gradient estimate


(12) $$\begin{array}{l} \;\nabla_{\theta }Ĵ\left(\pi_{\theta }\right)= \end{array}$$

$$\begin{array}{l} \frac{1}{B}\sum_{b=0}^{B}\sum_{t=0}^{T_{b}}\nabla_{\theta }\log\pi_{\theta }\left(a_{t,b}|s_{t,b}\right){Â}_{\pi_{\theta }}\left(s_{t,b},a_{t,b}\right). \end{array}$$

for a batch of trajectories


$$\tau_{b}=\left(s_{0,b},a_{0,b},r_{0,b},s_{1,b},a_{1,b},r_{1,b}...,s_{T_{b}+1,b}\right)$$

with *b* = 0, 1, ..., *B*. To mitigate the fact that the policy *pi*_θ_sample__ under which the samples/trajectories were collected is not equal to the current policy π_θ_ the advantage estimate is weighted by the policy ratio


(13) $$\begin{array}{l} {\text{pr}}_{t}\left(\theta \right):=\frac{\pi_{\theta }\left(a_{t}|s_{t}\right)}{\pi_{\theta_{\text{sample}}}\left(a_{t}|s_{t}\right)}. \end{array}$$

Furthermore, to ensure that the new policy does not diverge too much from the old one, which improves the stability, the ratio pr_*t*_(θ) is clipped. This leads to the PPO surrogate loss


(14) $$\begin{array}{l} \;L^{\text{PPO}}(\pi_{\theta }):=\\ E_{\tau \sim \pi_{\theta }}\left(\sum_{t=0}^{T}\min\left[{\text{pr}}_{t}(\theta ){\hat{A}}_{\pi_{\theta }}(s_{t},a_{t}),(\text{clip}({\text{pr}}_{t}(\theta ),\epsilon ){\hat{A}}_{\pi_{\theta }}(s_{t},a_{t})\right]\right) \end{array}$$

with the clipping function defined as


(15) $$\text{clip}(x,\epsilon ):=\{\begin{array}{cc} 1-\epsilon & x<1-\epsilon \\ x & 1+\epsilon \leq x\leq 1-\epsilon \\ 1+\epsilon & 1+\epsilon <x \end{array}$$

and a clip rate ϵ ∈ (0, 1). A regularizer based on the entropy of the policy is used as an exploration bonus to encourage the exploration of unseen states. Furthermore, the method works analogously for observations *o*_*t*_ instead of states *s*_*t*_, since they only lead to changes in the part estimated by samples and are thus automatically taken into account. A more detailed explanation about PPO can be found in the original paper Schulman et al. (2017) and about RL in general e.g. in Sutton and Barto (2018).

###  Probes

Probes, a technique from the mechanistic explainability area of AI, are utilized to analyze deep neural networks (Alain and Bengio, 2018). They are commonly applied in the field of natural language processing (Belinkov, 2022). Probes are typically small, neural network-based classifiers, usually implemented as shallow fully connected networks. They are trained on the activations of specific neurons or layers of a larger neural network to predict certain features, which are generally believed to be necessary or beneficial for the network's task. If probes achieve accuracy higher than chance, it suggests that the information about the feature, or something correlated to it, is present in the activations.

###  Implementation

We trained our agents in the NetHack environment using the NetHack Learning Environment (NLE) and MiniHack, a sandbox editor for custom scenarios in NetHack (Küttler et al., 2020; Samvelyan et al., 2021). NetHack provides a complex, discrete environment with low computational cost. It was first used as a benchmark at the NeurIPS 2021 NetHack challenge, where symbolic methods led by a wide margin (Hambro et al., 2022). Subsequently, NLE and MiniHack have been used for benchmarking reward modeling with large language model feedback (Klissarov et al., 2023), automatic curriculum design (Parker-Holder et al., 2022), internet query usage (Nottingham et al., 2022), skill transfer (Matthews et al., 2022), and planning with graph-based deep RL (Chester et al., 2022). It is also part of a benchmark platform for continual RL (Powers et al., 2022).

**Figure 4:**
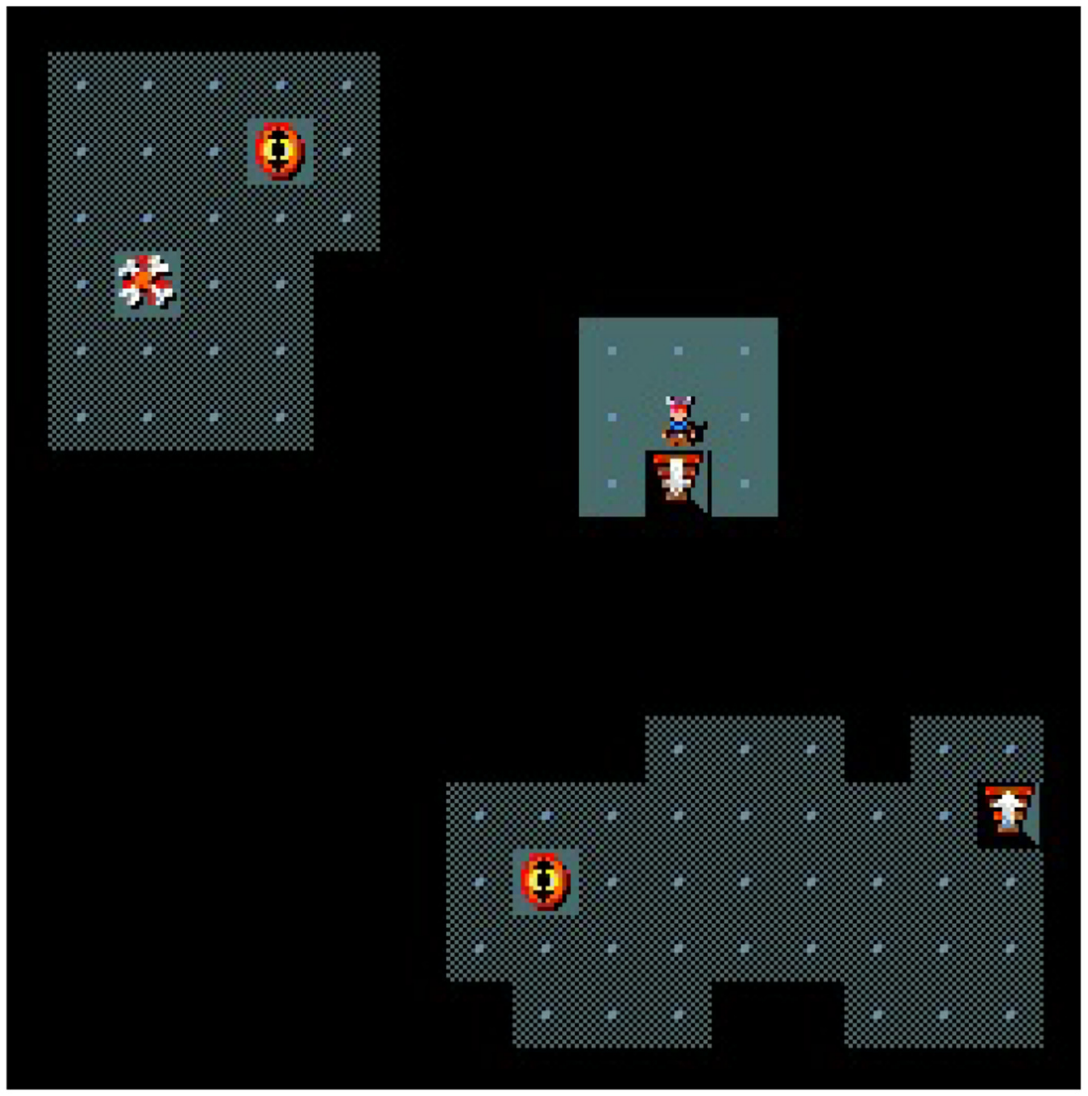
An example of the ultimate type map. The small figure is the agent, the staircases up and down are the start and goal, respectively, the eyes show uncovered teleportation traps and the bones are remains of a defeated monster. The dark gray areas have already been visited by the agent and the light gray 3 × 3 crop around the agent is the area he just discovered.

In this paper, we use the MiniHack-Room-Random-15x15-v0 (random), MiniHack-Room-Monster-15x15-v0 (monster), MiniHack-Room-Trap-15x15-v0 (trap), and MiniHack-Room-Ultimate-15x15-v0 (ultimate) maps from MiniHack. The random map consists of a 15 × 15 grid room with random start (staircase up) and goal (staircase down) positions. The monster map adds 3 monsters, and the trap map adds 15 teleportation traps. The ultimate map includes both features and is unlit, limiting the agent's view to a 3 × 3 window centered on itself (compare Figure 4). Teleportation traps are invisible until activated and move the agent to a random free location. All entities and monsters are randomly placed.

The agent can move in all cardinal and ordinal directions and observes the entire map and a 9 × 9 centered crop as glyphs, unique IDs for every game entity. Later, the action space was restricted to cardinal directions and observations to a 5 × 5 or 3 × 3 centered crop. Rewards are given as +1 for reaching the goal and −0.001 per step taken, with a maximum episode length of 300. Further details are in the MiniHack paper (Samvelyan et al., 2021).

###  Architecture of the agent

**Figure 5:**
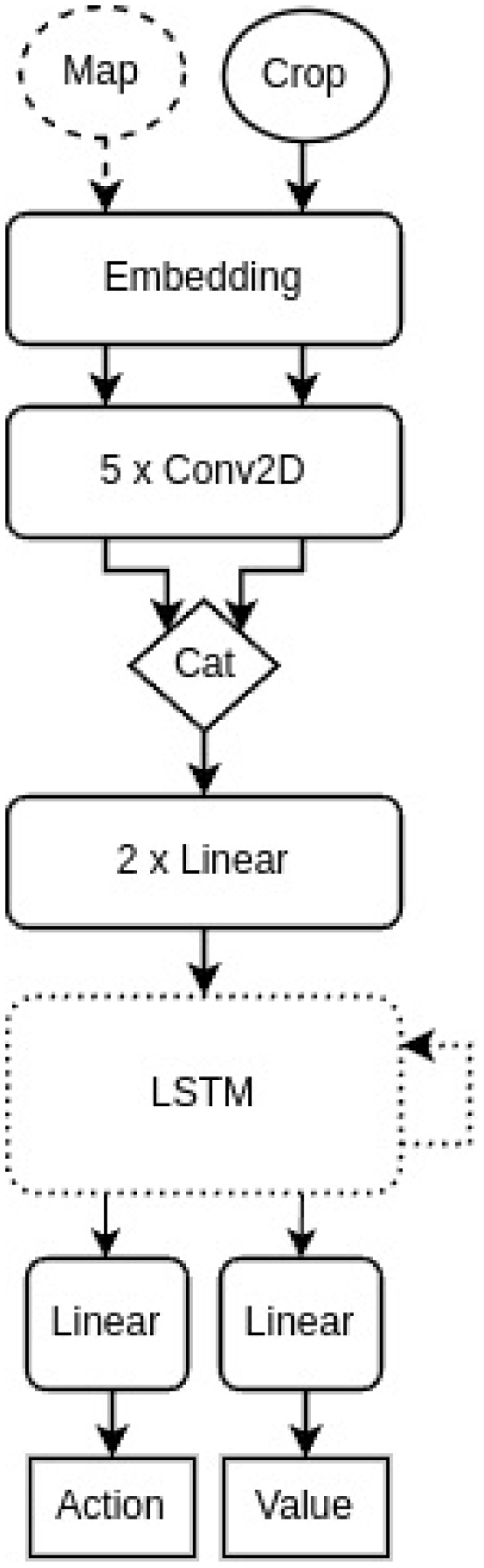
A simplified illustration of the agent architecture. The map (dashed circle) was only included as input in the first experiment and the LSTM cell (dotted rounded rectangle) became part of the architecture from the second experiment onward.

The basic agent architecture is a simplified version of their baseline model without Long Short-Term Memory (LSTM) cell and the parts to process the message and bottom line status as input e.g. the whole map and a centered crop of the map is given as input, the processing is done by an embedding layer, 5 Conv2D layers and 2 Linear layers followed by two parallel Linear layers with an action (distribution) and the estimated value function of the current state as final output, respectively. The embedding dimension was chosen as 64, each convolution layer contains 16 filters of size 3 besides the last one having only 8 filters. The hidden dimension of the linear layers is 256. From the second experiment onwards the LSTM cell was added back between the 2 Linear layers and the action and value heads. The cell and hidden state size was chosen as 512. The architectures are illustrated in Figure 5.

###  Training

RLlib (Liang et al., 2018) was used as the RL training framework and the agents were trained with their PPO implementation until convergence. The hyperparameters have been chosen similar to those recommended in the Minihack environment. An agent was trained only on a single map without any restriction of the random seeds.

## Results

Subsequent investigations employed probes to infer the spatial coordinates of the agent within the environment, predicated on the neural activations from a single network layer. For this purpose, a comprehensive dataset was compiled, encompassing 230, 000 instances per trained agent. These instances were derived by operationalizing the agent within its respective environment, during which both neural activations and corresponding spatial coordinates were documented. The dataset was apportioned into 200, 000 samples for training and 30, 000 for testing purposes. Training of the probes was oriented toward generating a predictive score for each possible coordinate across a 15 × 15 grid (both x and y axes). During the evaluation phase, the predicted coordinates with the highest scores were juxtaposed against the actual positions to assess the predictive accuracy of the probes.

In the initial experiment, a basic agent model without an LSTM cell was used to process inputs from the entire map and a localized 9 × 9 crop. The tests were conducted on two maps: the ultimate map and the trap map, using probes with a single linear layer. The results in Table 1 show mean accuracies exceeding random chance, especially on the ultimate map, suggesting that the agent's neural activations encode positional information. This pattern is stable across repeated probe trainings, with low standard deviations indicating consistent performance. However, the simplicity of the environment raises questions about whether this is due to direct observations or an internal model. This indicates the need for more complex architectures like RNNs or Transformers. Future experiments should train agents with an LSTM cell using a minimal central crop or use more intricate environments to prevent straightforward observation-based predictions.

**Table 1:** First experiment's results.

**Layer (output size) \map**	**Ultimate**	**Trap**
1. Conv2D (26544)	33.4 ± 0.92	8.8 ± 0.05
2. Conv2D (26544)	35.1 ± 0.34	8.8 ± 0.06
3. Conv2D (26544)	34.4 ± 0.17	8.7 ± 0.07
4. Conv2D (26544)	33.9 ± 0.37	8.7 ± 0.06
5. Conv2D (13272)	34.1 ± 0.15	8.8 ± 0.04
1. Linear (256)	28.1 ± 0.79	8.1 ± 0.24
2. Linear (256)	25.2 ± 1.27	8.0 ± 0.10

Mean accuracy and standard deviation (%) collected over 10 runs of the probes trained on the corresponding activations in predicting the correct x and y values of the position (separately). Chance is 6.7%. The basic agent architecture with map and 9 × 9 crop as input and without LSTM cell was used. The probes consist of one linear layer.

In the subsequent experiment, the agent architecture included a 5 × 5 crop as the sole input and an LSTM cell, tested across various maps. The agent's actions were limited to cardinal directions, and map edge positions were excluded to prevent deducing the agent's location from visual input. This setup emphasized the agent's ability to use historical data. Probes, both linear (single layer) and non-linear (three layers with ReLU activation), analyzed the memory aspect by training on the LSTM's hidden and cell states.

The results in Table 2 show mean accuracies exceeding chance, more so than in the first experiment, with low standard deviations indicating consistent performance across repeated probe trainings. Given the limited 5 × 5 crop input and exclusion of wall observations, positional information must come from the agent's memory and internal representations. Traps and monsters improved accuracies individually but not on the ultimate map, suggesting the need for more quantitative analysis to understand these effects.

**Table 2:** Second experiment's results.

**Input (type) \map**	**Random**	**Monster**	**Trap**	**Ultimate**
Hidden (linear)	25.6 ± 0.17	58.5 ± 0.22	40.6 ± 0.29	25.1 ± 0.08
Hidden (non-linear)	31.3 ± 0.19	64.0 ± 0.17	44.5 ± 0.43	27.7 ± 0.09
Cell (linear)	29.9 ± 0.25	62.2 ± 0.67	42.6 ± 0.68	26.5 ± 0.16
Cell (non-linear)	37.4 ± 0.17	67.4 ± 0.11	47.3 ± 0.31	30.4 ± 0.10

Mean accuracy and standard deviation (%) collected over 10 runs of the probes trained on the hidden and cell states of the LSTM cell in predicting the correct x and y values of the position (separately). Chance is 9.1%. Two rows on the left, right, top, and bottom of the map were excluded from the dataset. The agent architecture with only a 5 × 5 crop as input and including the LSTM cell with hidden/cell size 512 was used. The linear probes consist of one linear layer and the non-linear probes of 3 linear layers with ReLUs in between.

The third experiment mirrored the second, except the crop size was reduced to 3 × 3. Probes were trained for 50 epochs using the Adam optimization algorithm, with learning rates set to 0.00005 for linear probes in the first experiment, and 0.001 for linear and 0.0001 for non-linear probes in the second and third experiments. Each setting was run 10 times with unconstrained random seeds to estimate the mean and standard deviation. Increased training epochs and advanced learning strategies could potentially enhance performance.

The findings from the third experiment, shown in Table 3 corroborate prior results, with mean accuracies significantly above random chance and low standard deviations indicating consistent performance across repeated probe trainings. However, the highest accuracies were on the simplest random map, and the smaller 3 × 3 crop led to lower accuracies on the monster and trap maps. This indicates that a smaller observational input may hinder the agent's ability to accurately infer its position in complex environments.

**Table 3:** Third experiment's results.

**Input (type) \map**	**Random**	**Monster**	**Trap**	**Ultimate**
Hidden (linear)	54.8 ± 0.08	49.5 ± 0.08	34.0 ± 0.10	27.6 ± 0.16
Hidden (non-linear)	58.1 ± 0.30	53.3 ± 0.14	35.4 ± 0.15	28.9 ± 0.20
Cell (linear)	57.5 ± 0.30	50.3 ± 0.27	34.2 ± 0.20	28.3 ± 0.24
Cell (non-linear)	59.7 ± 0.16	54.7 ± 0.15	36.3 ± 0.12	30.4 ± 0.12

Mean accuracy and standard deviation (%) collected over 10 runs of the probes trained on the hidden and cell states of the LSTM cell in predicting the correct x and y values of the position (separately). Chance is 7.7%. One row on the left, right, top, and bottom of the map was excluded from the dataset. The agent architecture with only a 3 × 3 crop as input and including the LSTM cell with hidden/cell size 512 was used. The linear probes consist of one linear layer and the non-linear probes of 3 linear layers with ReLUs in between.

These results align with preliminary experiments that varied crop sizes, embedding dimensions, hidden/cell state sizes, and convolutional layers across different maps. Accuracies ranged from 17% to 67%, consistently above chance levels. The specific agent configuration and the baseline chance level also influenced the final accuracy outcomes.

## Discussion

###  Evidence of world model

Our initial findings suggest that the hidden layer activations encapsulate information regarding the agent's position. Nevertheless, given the simplistic nature of the environment, it remains ambiguous whether this information is directly extracted from observations or assimilated by the agent through a world model. To enhance the efficacy of the method, implementing a more expressive agent architecture, such as a recurrent neural network (RNN) or transformer, coupled with observations that provide less direct information (e.g., a centered crop), is essential.

Consequently, in our subsequent experiments, we trained agents equipped with an LSTM cell, utilizing a narrowly centered crop for observation. Alternatively, introducing a more complex environment could be considered. We also constrained the action space, anticipating that this limitation would foster simpler and more precise latent representations. The findings from our second and third experiments robustly confirm that the agent's position is encoded within the network's activations and indicate that the agent has developed a world model. To rigorously evaluate the impacts and influences of various architectures, environmental settings, and training methodologies, a more detailed and extensive quantitative study is required.

Reducing the crop size complicates the learning challenge but highlights the importance of the agent's ability to infer its position for efficient map navigation. Teleportation traps further obscure the agent's positional accuracy, indicating that lower accuracy might still reflect a more refined world model. A direct comparison of agents could be facilitated by evaluating all agents on a uniform random map, a logical progression for future research. Analysis suggests that the cell state may contain slightly more information than the hidden state, with a non-linear representation enhancing accuracies with non-linear probes. A more comprehensive investigation is needed to thoroughly understand these impacts. However, this paper provides evidence supporting the existence of a world model, with detailed exploration reserved for future studies.

###  Environment choice and possible extensions

The inclusion of teleportation traps and monsters in the virtual environment was an intentional design choice aimed at testing the robustness and memory dependence of the agent's internal representations. In particular, teleportation traps—which instantaneously relocate the agent to a random free position on the map—introduce discontinuities that violate standard assumptions of spatial and temporal coherence. While this mechanism may appear artificial compared to physical displacements in real-world environments, it serves as an effective stress test: the abrupt loss of positional continuity forces the agent to rely on internal memory and world modeling capabilities rather than immediate visual input. This allows us to assess whether the agent has formed a persistent, coherent representation of the environment or whether its behavior is strictly reactive. Similarly, the presence of monsters introduces dynamic elements that require agents to generalize across different local contexts and adapt to moving obstacles. In future experiments, we plan to compare these results with setups involving structured displacements or more biologically plausible perturbations to better understand their differential impact on the development of self and world models.

We acknowledge that the environments used in our experiments are intentionally simplified, with limited spatial structure and relatively constrained task complexity. This design choice was made to enable controlled probing of the agent's internal representations, allowing us to isolate whether and how self- and world models emerge under basic reinforcement learning conditions. Simple environments help reduce confounding factors and allow for clearer interpretation of results, particularly when assessing the influence of architecture, memory, and observational scope on positional encoding. However, we agree that such settings do not fully capture the challenges of more realistic or dynamic contexts. Future work will extend these experiments to richer environments featuring complex spatial topologies, delayed rewards, stochastic dynamics, and possibly multi-agent interaction, thereby testing whether the emergence and robustness of internal models scale with environmental complexity. Diverse challenges, and multifaceted objectives—such as long-term planning, problem-solving, social interactions, and adaptive learning—will rigorously test agents' potential for consciousness. This will improve evaluation of their ability to form complex internal representations and understand the scalability and generalizability of the models, enhancing the reliability of the findings.

Extending model evaluations to various environments or real-world scenarios is essential for assessing generalizability. While current virtual environments provide controlled settings, real-world applications are more complex. Testing agents in settings like real-time robotics, social simulations, and dynamic ecological systems will offer a comprehensive understanding of their cognitive and adaptive capabilities. This broader evaluation will determine if insights from virtual environments apply to practical scenarios, enhancing relevance and identifying areas for improvement, guiding the development of more robust AI systems.

###  From LSTMs to more advanced architectures

Exploring advanced architectures like transformers and sophisticated RNNs holds potential for investigating mechanisms relevant to machine consciousness. While the current architectures are sufficient for initial experiments, they may fall short in modeling the temporal depth and structural complexity required for higher-order cognitive processes. Transformers, with their ability to capture long-range dependencies and parallelize training, and advanced RNNs, which offer improved handling of sequential dynamics, may enable the emergence of more stable and expressive internal models. These capabilities could facilitate more nuanced self- and world modeling, providing a richer foundation for studying how artificial agents form and exploit internal representations—an essential step toward probing the computational prerequisites for consciousness-like functionality.

###  Relation between consciousness and intelligence

While increasing the complexity of an agent's environment and architecture often fosters the development of more advanced cognitive capacities—such as planning, generalization, or long-term memory use—it is important to emphasize that this does not necessarily imply a corresponding increase in consciousness. Intelligence and consciousness are categorically distinct constructs: intelligence refers to an agent's ability to solve problems and adapt effectively to its environment, whereas consciousness involves the presence of integrated, subjective experience or self-referential processing. Our work does not assume a direct or causal relationship between these domains. Rather, we use complex environments as experimental scaffolds to assess whether the structural or functional precursors for consciousness—as articulated in Damasio's theory—can emerge under more demanding conditions. Any further inference about consciousness from observed intelligence must be supported by both empirical evidence and theoretical justification, not presumed based on task performance alone.

###  Self vs. world models

An agent's ability to discern its position may suggest basic core consciousness, but this is not conclusive. Differentiating between a world model and a self model is crucial. According to Damasio, a self model is based on stable internal sensations, while a world model relies on variable external observations (Damasio and Meyer, 2009). However, Damasio and Damasio (2022) focuses on differentiating homeostatic feelings and external inputs, which may not directly address internal vs. external models.

Future research will have to distinguish between self and world models to advance machine consciousness understanding. In addition to an agent consistently appearing in the middle of a crop as a stable element while other fields are chaotic, this might involve incorporating internal state variables into the agent's input and reward structure to observe how these fluctuations affect behavior and decision-making. This will reveal if an agent can develop a true self model, characterized by stable internal sensations, distinct from variable external observations forming the world model. Analyzing the interplay between these representations is crucial for validating core consciousness per Damasio's framework, laying the groundwork for more sophisticated AI systems.

A key direction for future research lies in moving beyond the detection of internal models toward evaluating their functional and causal role in agent behavior. Specifically, the ability to actively exploit internal representations—such as those encoding position, goals, or internal state variables—to guide decisions is more closely aligned with notions of agency and Damasio's concept of a functional self. For agents with an interpretable world model, one promising approach is to systematically intervene on neural activations—e.g., perturbing units correlated with spatial localization or internal “feelings”—and observe whether such manipulations alter the agent's policy or trajectory in context-sensitive ways. For agents with an interpretable world model, modifying specific neural activations—such as those representing the agent's position—not only allows for examining their influence on actions, but also provides a more rigorous test of whether internal models are actively and intentionally used, aligning with methods in Li et al. (2022). Such experiments would offer a stronger foundation for evaluating the emergence of goal-directed control and self modeling, thereby contributing to a deeper computational understanding of proto-consciousness and machine agency.

###  Limitations

It is important to emphasize that our findings should not be interpreted as evidence for the instantiation of consciousness in artificial agents. Rather, our work explores whether structural and functional precursors—such as integrated self- and world models grounded in internal state representations—can emerge within standard reinforcement learning frameworks. These features are modeled after the components that Damasio's theory identifies as necessary for core consciousness, but their presence does not imply the existence of subjective experience. What we observe are simulations or functional analogs of conscious processes, not consciousness itself. We adopt a deliberately cautious stance: our goal is to investigate whether architectures and learning dynamics in artificial systems can give rise to mechanisms that resemble those found in theories of consciousness—not to claim that such systems possess phenomenality or sentience. This distinction between simulating and instantiating consciousness is critical, and it frames our contribution as a step toward mechanistic insight, not metaphysical assertion.

In line with Damasio's theory, we frame the development of internal world and self models as necessary—but not sufficient—conditions for the emergence of core consciousness. Our experimental setup investigates whether such structures can arise in artificial agents as a byproduct of reinforcement learning, particularly through interactions that simulate basic homeostatic regulation and environmental navigation. Specifically, Damasio's concept of core consciousness involves the dynamic mapping of the relationship between a protoself (a representation of internal bodily states) and a changing world, resulting in a transient sense of self situated in context. In our implementation, the agent's self model is implicitly formed through memory representations of internal state trajectories (e.g., hidden and cell states in the LSTM), while the world model is approximated by the agent's learned ability to encode and predict spatial features of the environment. However, we acknowledge that critical components of Damasio's framework—such as complex affective feedback, fine-grained interoception, and higher-order autobiographical memory—are not fully captured. Our goal is not to claim completeness, but to show that structurally relevant precursors to core consciousness, as defined by this theory, can begin to emerge under suitable training conditions in artificial systems.

It is worth noting that Damasio's terminology—such as proto-self, core consciousness, and extended consciousness may be seen by some critics as a rebranding of well-established neurocognitive functions, including bodily state representation, perception-action integration, and executive processes like memory and planning. However, the value of Damasio's framework lies in how it organizes these functions into a coherent, hierarchically structured model of consciousness. This process-oriented narrative facilitates not only theoretical interpretation but also practical implementation in artificial agents. In our work, we adopt this terminology not as a literal assertion of consciousness in machines, but as a conceptual scaffold for operationalizing and probing the emergence of self- and world models. Framing these internal structures through Damasio's lens enables the formulation of testable hypotheses about how such representations might arise through interaction with a virtual environment.

A well-known point of contention in Damasio's theory concerns the relationship between neural representations of emotional states—termed “feelings”—and the emergence of subjective experience. While the theory posits that consciousness arises in part from the internal mapping of bodily-emotional states, it remains philosophically and scientifically unresolved how or why such representations would yield phenomenological awareness. This explanatory gap has been criticized as leaving the hard problem of consciousness intact. Nonetheless, from a computational standpoint, the distinction between emotions (as reactive behavioral programs) and feelings (as internalized representations of those reactions) offers a useful functional architecture. In artificial agents, this mapping allows for the modeling of self-monitoring mechanisms that respond to changes in internal or environmental states. Even without invoking subjective experience, this layered structure supports testable hypotheses about how agents might regulate behavior, adapt to uncertainty, or form persistent self-world representations—core components in the operational study of machine consciousness.

Our current approach treats scalar reward signals as a simplified proxy for affective states, aligning with the basic reinforcement learning framework in which rewards guide adaptive behavior. However, we acknowledge that this reductionist mapping does not capture the full richness or diversity of biological feelings. In affective neuroscience, emotional experience is often modeled either categorically—as distinct systems such as seeking, fear, rage, and play (Panksepp, 2004; Solms, 2021)—or dimensionally, across axes such as valence and arousal. Both perspectives suggest that real emotional states involve multiple interacting components beyond a single numerical value. While scalar rewards may approximate rudimentary signals of homeostatic success or failure, modeling more complex emotional architectures in artificial agents would likely require multiple internal state variables, competing motivational systems, and structured feedback loops. Future work could explore such multi-dimensional frameworks to better emulate the functional diversity of affective processes and investigate their role in the emergence of self models and adaptive behavior. Furthermore, agents should be trained with inputs related to physiological states, like hitpoints or experience levels, using changes in these inputs as rewards.

While our use of reward signals as proxies for emotions aligns with Damasio's functional distinction between emotions (reactive behaviors) and feelings (internal representations of those behaviors), it is essential to acknowledge a fundamental difference between biological and artificial agents. In living organisms, emotions are tightly coupled to survival and homeostatic regulation; they reflect evaluative attitudes shaped by evolutionary pressures and are experienced as meaningful because the organism has a stake in the outcome. Artificial agents, by contrast, operate within externally defined reward structures and lack any intrinsic motivation or existential concern. The reward signals they receive carry no inherent significance beyond their instrumental role in optimizing behavior. Thus, our implementation should be understood as a heuristic framework—one that enables the modeling of internal state monitoring and adaptation, but without implying any ontological equivalence to biological emotion. This distinction is central to any rigorous inquiry into artificial consciousness, and we emphasize that our goal is to explore functional architectures, not to claim artificial sentience.

It is important to emphasize as well that our study does not claim to model or detect subjective experience in artificial agents. Instead, we focus on probing whether structural and functional elements identified by Damasio as prerequisites for core consciousness—such as the integration of self- and world models grounded in affect-like internal signals—can emerge through reinforcement learning. This approach reflects a theoretical stance rather than a metaphysical one: we adopt Damasio's framework not because it offers a definitive account of consciousness, but because it provides a structured, biologically informed model that can be operationalized in artificial systems. Compared to alternative theories such as IIT, which demands a high-resolution causal analysis, or GWT, which presupposes symbolic broadcasting and attentional architectures, Damasio's model is more readily applicable to embodied agents interacting with dynamic environments. That said, we fully acknowledge the diversity of views in the consciousness literature and view our work as a computational instantiation of one such perspective—not a claim of theoretical superiority. Future research should explore similar operationalizations of alternative theories to better understand their respective implications for machine-based models of consciousness.

The relationship between neural representations and consciousness remains a central topic in theoretical neuroscience and philosophy of mind. According to Seth and Bayne (2022), consciousness is not merely correlated with the presence of neural representations, but may depend on specific representational structures—such as integrated, multimodal content that is accessible for cognitive use. Similarly, Kuhn's landscape of consciousness framework (Kuhn, 2024) categorizes theories of consciousness based on how they interpret the role of neural encoding, from purely information-theoretic to biologically embodied models. Within Damasio's theory, core consciousness emerges not simply from the presence of representations, but from their integration—particularly of self- and world-related content—and their modulation by homeostatic and emotional signals. In our study, we adopt this view by treating internal representations as functionally meaningful substrates that may support core consciousness when sufficiently structured and coupled to behavior. While we do not claim that these representations instantiate phenomenality, their emergence within reinforcement learning agents offers a testable proxy for assessing the development of consciousness-relevant internal architecture.

While Damasio's theory emphasizes the emergence of core consciousness through ongoing interactions between self and environment, it does not reduce consciousness to purely reactive processes. In his broader framework, extended consciousness encompasses a range of internally driven, endogenous functions such as autobiographical memory, planning, imagination, and internal narrative construction. These processes allow conscious experience to persist even in the absence of immediate external stimuli. This view aligns with neuroscientific findings on spontaneous brain activity, such as those observed in resting-state networks, which support internally generated thought and perception (Raichle et al., 2001; Christoff et al., 2016). Although our current experiments focus on externally oriented, task-driven behavior to probe for structural elements of core consciousness, future work could incorporate memory systems, generative models, or predictive coding mechanisms to explore how artificial agents might simulate or generate internal experiences independent of immediate sensory input.

While our study focuses on the emergence and interaction of self- and world models—key components of Damasio's conception of core consciousness—it is important to note that such integration may already correspond to a relatively advanced stage within the broader spectrum of conscious phenomena. More rudimentary or “selfless” forms of consciousness, such as raw sensory registration, momentary arousal, or affective salience without self-referential modeling, may precede the development of a distinct core self. Damasio's own framework accommodates this gradation, beginning with the protoself and ascending through increasingly complex layers of conscious processing. Our experimental focus on position inference and memory-based representation reflects an intermediate level where self-related information is actively modeled and used. We acknowledge that this excludes simpler conscious states and reinforces the need for graded, theory-informed approaches when evaluating artificial systems for consciousness-like properties.

## Conclusion

Our results show that constructing an internal model is crucial for efficiently solving certain tasks. This suggests that even model-free reinforcement learning approaches might develop implicit internal models, making them not truly model-free. Additionally, some RL exploration strategies require predictions about environmental dynamics, highlighting the practicality of such models. Using this internal model for exploration could further integrate it into the agent's functionality. In our approach, we used a discount factor for RL to ensure mathematical convergence over an infinite horizon by reducing the impact of distant rewards and emphasizing immediate rewards, effectively modeling future uncertainty. Remarkably, in the context of successor representations (SR) (Stachenfeld et al., 2017; Gershman, 2018), the discount factor plays a similar role by determining how much future states influence the representation of the current state. In particular, in SR the discount factor adjusts the expected discounted future state occupancy, shaping the cognitive map of the environment enabling agents (including humans and animals) to plan and make decisions based on their expectations of future states (Stoewer et al., 2022, 2023c,a,b; Surendra et al., 2023). This concept bridges reinforcement learning theories with cognitive science, providing insights into how intelligent behavior emerges from the interaction with the environment. In particular, SR can be seen as a bridge between model-free and model-based approaches (Momennejad et al., 2017; Botvinick et al., 2019). Cognitive maps may offer a possible way to more directly simulate world and self models.

Beyond its theoretical appeal, Damasio's model of consciousness offers practical insights that can inform the design of reinforcement learning systems. Structuring agents in alignment with the model's hierarchical architecture—protoself, core consciousness, and extended consciousness—suggests a pathway for integrating layered internal representations that reflect bodily state, situated experience, and memory-based reasoning. Such a framework can introduce beneficial inductive biases for RL: self models may promote interpretability and modularity in policy learning, while world models can support counterfactual reasoning and planning. Moreover, Damasio's emphasis on homeostatic regulation naturally aligns with current approaches in intrinsic motivation and curiosity-driven exploration, where internal state dynamics shape the learning signal. Embedding these principles into agent design could lead to more robust learning in sparse or deceptive environments, and may enhance adaptability by fostering agents that learn to self-monitor and maintain internal equilibrium. Thus, while our current work probes whether such structures emerge spontaneously, Damasio's theory also provides computational heuristics for developing next-generation RL architectures.

Our study underscores the capabilities of AI methodologies, particularly RL, in exploring theories of consciousness and advancing explainable AI. Considering AI consciousness is crucial for understanding AI's goal-directed behavior and ensuring AI safety. While unconscious AIs can impact outcomes, their lack of awareness means they can't be classified as friendly or evil. Exploring AI consciousness is vital for evaluating the risks and opportunities in AI. Through this work, we aim to contribute to the discourse on whether machines can achieve consciousness.

## Data Availability

The raw data supporting the conclusions of this article will be made available by the authors, without undue reservation.

## Footnotes

^1^In the context of this study, we define a neural representation as a pattern of activity across a population of units (biological neurons or artificial nodes) that systematically encodes information about an internal or external feature of the environment. Such representations allow an agent to retain, manipulate, or simulate relevant aspects of the world, including its own internal state, even when those features are not currently observable. This concept aligns with established views in neuroscience and cognitive science, where neural representations are considered foundational for perception, memory, and decision-making processes (Kriegeskorte et al., 2012).

^2^By “essential structures and dynamics,” we refer to regularities in the environment that are critical for competent agent behavior. These include spatial layouts (e.g., room geometry or object locations), transition patterns (e.g., how specific actions affect the agent's state), and interaction contingencies (e.g., consequences of collisions, rewards for reaching goals). For an internal representation to be considered meaningful, it must capture these underlying patterns in a way that enables the agent to predict outcomes, adapt to changes, and make informed decisions based on partial observations. In our experiments, the ability to localize oneself or infer future states from limited visual input and memory serves as an operational indicator of whether such structural regularities have been internalized.
